# Supplementary material for: Endorsement of the 32-item Consolidated Criteria for Reporting Qualitative (COREQ) and Standards for Reporting Qualitative Research (SRQR) by Chinese journals of nursing: A survey of editors and review of journal instructions for authors
Source: PLoS One. 2026 Apr 22;21(4):e0346938. doi: 10.1371/journal.pone.0346938 (PMC13102231; doi:10.1371/journal.pone.0346938)
Supplement: S1 File — (DOCX) [file pone.0346938.s001.docx]

**S1 File** Basic information of the Chinese nursing journals included in this study.

| Journal Name | Database | Language | ISSN | Website |
| --- | --- | --- | --- | --- |
| Chinese Journal of Nursing | CSCD | Chinese | 0254-1769 | http://zh.zhhlzzs.com |
| Chinese Journal of Nursing Education | CSCD | Chinese | 1672-9234 | http://jy.zhhlzzs.com |
| Chinese Nursing Management | CSCD | Chinese | 1672-1756 | http://www.zghlgl.com |
| Military Nursing | CSCD | Chinese | 2097-1826 | http://cpnj.smmu.edu.cn |
| Journal of Nursing Science | CSCD | Chinese | 1001-4152 | http://www.hlxzz.com.cn |
| Chinese Journal of Emergency and Critical Care Nursing | CSCD | Chinese | 2096-7446 | http://jwzz.zhhlzzs.com |
| Journal of Nursing Administration | CSCD | Chinese | 1671-315X | http://www.huliguanlizazhi.com |
| International Journal of Nursing Sciences | CSCD | English | 2352-0132 | http://www.zhhlzzs.com |
| Chinese Nursing Research | CSTPCD | Chinese | 1009-6493 | http://www.hlyjzz.com |
| Journal of Nursing(China) | CSTPCD | Chinese | 1008-9969 | http://hlxbzz.soripan.net |
| Modern Clinical Nursing | CSTPCD | Chinese | 1671-8283 | http://xdlchl.boyuancb.com |
| Chinese Journal of Modern Nursing | CSTPCD | Chinese | 1674-2907 | http://zhxdhlzz.yiigle.com |
| Journal of Nurses Training | CSTPCD | Chinese | 1002-6975 | https://fsjx.cbpt.cnki.net |
| Chinese Journal of Practical Nursing | CSTPCD | Chinese | 1672-7088 | http://www.zgsyhlzz.com |
| Modern Nurse | Others | Chinese | 1006-6411 | http://www.ddhszz.com |
| Chinese Clinical Nursing | Others | Chinese | 1674-3768 | http://journal16.magtechjournal.com |
| Medical Higher Vocational Education and Modern Nursing | Others | Chinese | 2096-501X | https://gzyhl.jssmu.edu.cn |
| Nursing of Integrated Traditional Chinese and Western Medicine | Others | Chinese/English | 2096-0867 | www.zxyjhhl.com.cn |
| Journal of Clinical Nursing | Others | Chinese | 1671-8933 | http://www.lchlzzs.com |
| Chinese Evidence-Based Nursing | Others | Chinese | 2095-8668 | http://www.xzhlzz.com |
| Chinese General Practice Nursing | Others | Chinese | 1674-4748 | http://www.qkhlzz.com |

**S1 File** Basic information of the Chinese nursing journals included in this study. (continued)

| Journal Name | Database | Language | ISSN | Website |
| --- | --- | --- | --- | --- |
| Shanghai Nursing | Others | Chinese | 1009-8399 | http://shhl.ijournal.cn |
| Journal of Nursing and Rehabilitation | Others | Chinese | 1671-9875 | http://www.zjhlykf.com |
| Journal of Qilu Nursing | Others | Chinese | 1006-7256 | http://www.qlhlzz.net |
| International Journal of Nursing | Others | Chinese | 1673-4351 | http://gjhlxzz.yiigle.com |
| Nursing Practice and Research | Others | Chinese | 1672-9676 | http://www.hlsjyj.com |
| Tianjin Journal of Nursing | Others | Chinese | 1006-9143 | http://www.tjhlbjb.com |
| Electronic Journal of Practical Clinical Nursing Science | Others | Chinese | 2096-2479 | http://sylchlxdzzz.soripan.net |
| Frontiers of Nursing | Others | English | 2095-7718 | http://www.hlqyzz.com |
